# Quantitative spatio-temporal characterization of epileptic spikes using high density EEG: Differences between NREM sleep and REM sleep

**DOI:** 10.1038/s41598-020-58612-4

**Published:** 2020-02-03

**Authors:** Xuan Kang, Melanie Boly, Graham Findlay, Benjamin Jones, Klevest Gjini, Rama Maganti, Aaron F. Struck

**Affiliations:** 1 University of Wisconsin-Madison Department of Neurology, Madison, Wisconsin 53705 USA; 2 University of Wisconsin-Madison Department of Psychiatry, Madison, Wisconsin 53705 USA

**Keywords:** Predictive markers, Epilepsy

## Abstract

In this study, we applied high-density EEG recordings (HD-EEG) to quantitatively characterize the fine-grained spatiotemporal distribution of inter-ictal epileptiform discharges (IEDs) across different sleep stages. We quantified differences in spatial extent and duration of IEDs at the scalp and cortical levels using HD-EEG source-localization, during non-rapid eye movement (NREM) sleep and rapid eye movement (REM) sleep, in six medication-refractory focal epilepsy patients during epilepsy monitoring unit admission. Statistical analyses were performed at single subject level and group level across different sleep stages for duration and distribution of IEDs. Tests were corrected for multiple comparisons across all channels and time points. Compared to NREM sleep, IEDs during REM sleep were of significantly shorter duration and spatially more restricted. Compared to NREM sleep, IEDs location in REM sleep also showed a higher concordance with electrographic ictal onset zone from scalp EEG recording. This study supports the localizing value of REM IEDs over NREM IEDs and suggests that HD-EEG may be of clinical utility in epilepsy surgery work-up.

## Introduction

Epilepsy affects over 60 million individuals worldwide and carries significant risks of severe injury and sudden death^1^. The majority of mortality and morbidity occurs in 30–40% of patients with medication refractory epilepsy^2^. The presence of epileptic spikes has also been correlated with negative cognitive consequences^3,4^. Epilepsy surgery has been shown to be an effective treatment for medication refractory patients, if the epileptogenic zone (EZ) can be correctly identified^5^. The goal of epileptic surgery is also to resect the EZ while preserving functional brain areas. Currently, the identification of the ictal onset zone through triggering seizures in the epilepsy monitoring unit remains the gold standard to localize the EZ. However, routine EEG has limited spatial resolution, and seizures cannot always be captured. As a result, a focal delineation of the EZ from inter-ictal recordings is desirable.

EEG remains the most important clinical technique for epilepsy characterization and presurgical work up^6^. In particular, the study of inter-ictal epileptiform discharges (IEDs) is used to localize a cortical irritative zone, which often overlaps with the EZ. Over the last few years, high-density EEG has been increasingly used to localize IEDs. Indeed, traditional 10–20 scalp EEG is unable to localize IEDs or seizure onset zone with sufficient resolution for surgery^7–11^. Invasive EEG (iEEG) could in principle provide more accurate localization for EZ^12,13^, but invasive studies are inherently limited by spatial sampling and cannot cover the whole brain. Their success depends on hypotheses developed from non-invasive studies. EEG source imaging (ESI) techniques are alternative methods that provide more accurate location of the cortical generators of the EEG signals compared to 10–20 clinical EEG studies, while remaining non-invasive^14,15^. Accurate source localization using ESI requires the use of high-density EEG (HD-EEG), as the accuracy of mapping of the EZ increases monotonically with the number of electrodes used^11,14,16^. While HD-EEG studies have been shown to predict surgical outcomes^17^ and IEDs are known to be most frequent during sleep, little is known about differences in accuracy resulting from source reconstruction of IEDs captured during different stages of sleep.

It has been long suspected that sleep stage may have an effect on the spatio-temporal distribution of IEDs^18–20^. Case reports have suggested that IEDs during rapid eye movement (REM) sleep, instead of non-rapid eye movement (NREM) sleep, provide more precise localization regarding EZ^21,22^. However, group studies using HD-EEG are needed to quantify systematic differences and correlate ESI results with other EZ markers. HD-EEG topographies and source-localization techniques improve spatial resolution and make quantitative comparison between IEDs across sleep stages possible, which may have a direct implication for epilepsy surgery work up.

As such, the objective of this study is to characterize and compare IEDs during REM sleep and NREM sleep in scalp HD-EEG recordings and after applying ESI in patients with refractory focal epilepsy. We attempt to characterize the duration and spatial involvement of IEDs, both at the scalp level and in source space during REM sleep and NREM sleep. All results presented are corrected for multiple comparisons in space and time and described both at the group level and at the individual subject level.

## Result

### Patient demographics

Out of the fifteen patients, six had spikes during both REM sleep and NREM sleep. Demographics for these six patients, including clinical EEG and imaging results, are displayed in Table 1. Number of spikes during REM ranged from 1 to 10 [mean number of spikes + standard deviation (s.d.); 4 + 3] and during NREM sleep ranged from 31 to 41 [mean number of spikes + standard deviation (s.d.); 35 + 4] (See Supplementary Table S1).

**Table 1:** Characteristics of each subject.

ID	Age/gender	Ictal Onset Zone	Inter-ictals	Antiepileptic medications	Imaging studies
P01	64/F	Left temporal	Bi-temporal lobe slowing	Levetiracetam, Lacosamide	Normal MRI
P02	66/M	Left frontal	Left frontal and temporal spikes	Levetiracetam, Zonisomide	Normal MRI
P03	64/F	Left temporal	Left temporal slowing or sharps	VNS, carbamazepine, Zonisomide	MRI: left MTS; PET: left temporal hypo-metabolism
P04	34/F	Right parietal	Bilateral posterior quadrant spikes	Lamotrigine, Topiramate, Pregabalin	MRI: Bilateral grey matter heterotopia at temporal horn.
P05	37/M	Left frontal	Left temporal spikes	Clonazepam, Lacosamide, Lamotrigine, Levetiracetam	MRI: right temporal lesion. PET: Hypo-metabolism of R Temporal lobe
P06	32/F	Left frontal	Left fronto-central epileptiform activities.	Clonazepam Lamotrigine, Levetiracetam	Normal MRI

MTS: Mesial Temporal Sclerosis; PET: Position Emission Tomography; MRI: Magnetic resonance imaging; VNS: Vagus Nerve stimulator; F: female; M: male; EEG: Electroencephalogram.

*The terms “Ictal onset zone’ and “Inter-ictals” refers to the location of seizure onset zone and interictal epileptiform discharges captured on clinical 10–20 EEG during the same EMU admission.

### Scalp level analysis

**Figure 1:**
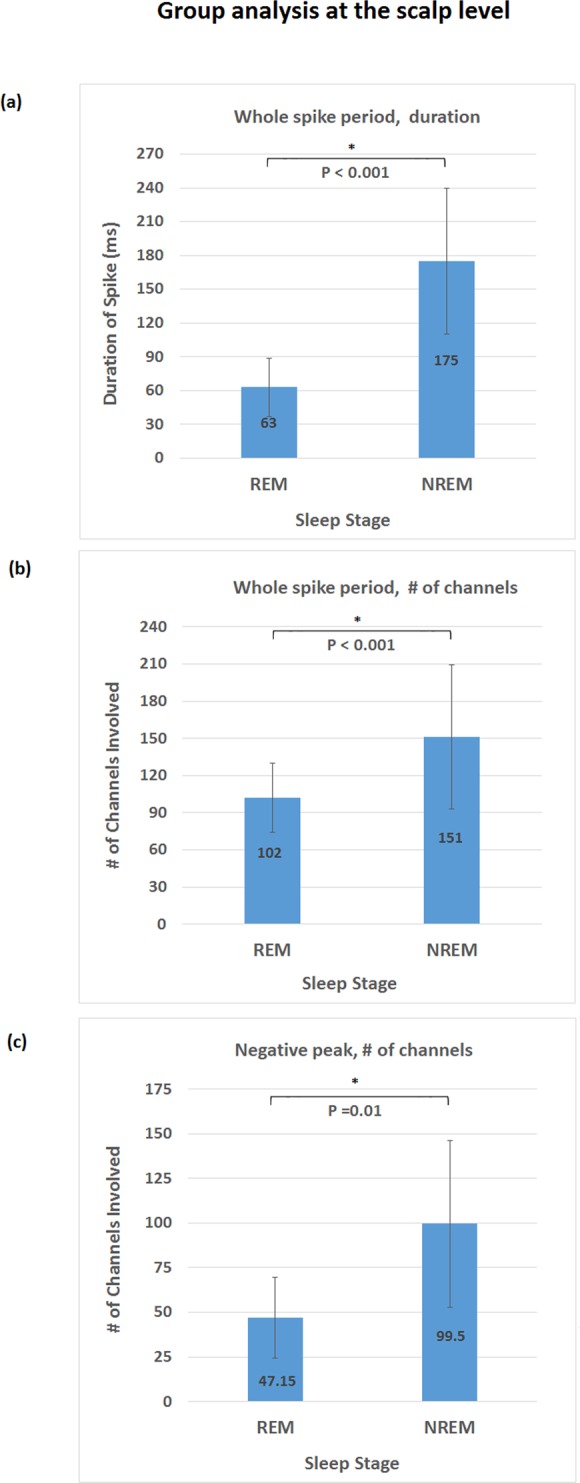
Group level analysis at the scalp level: asterisk is applied for result that reached statistical significance. (**a**) IED duration based on analysis of the whole spiking period, with shorter duration (p = 5 × 10^−11^) during REM sleep. (**b**) Number of channels involved during IED based on analysis of the whole spiking period, with less channels involved (p = 2 × 10^−5^) during REM sleep. (**c**) Number of channels involved at the negative peak, with less channels involved (p = 0.01) during REM sleep.

**Figure 2:**
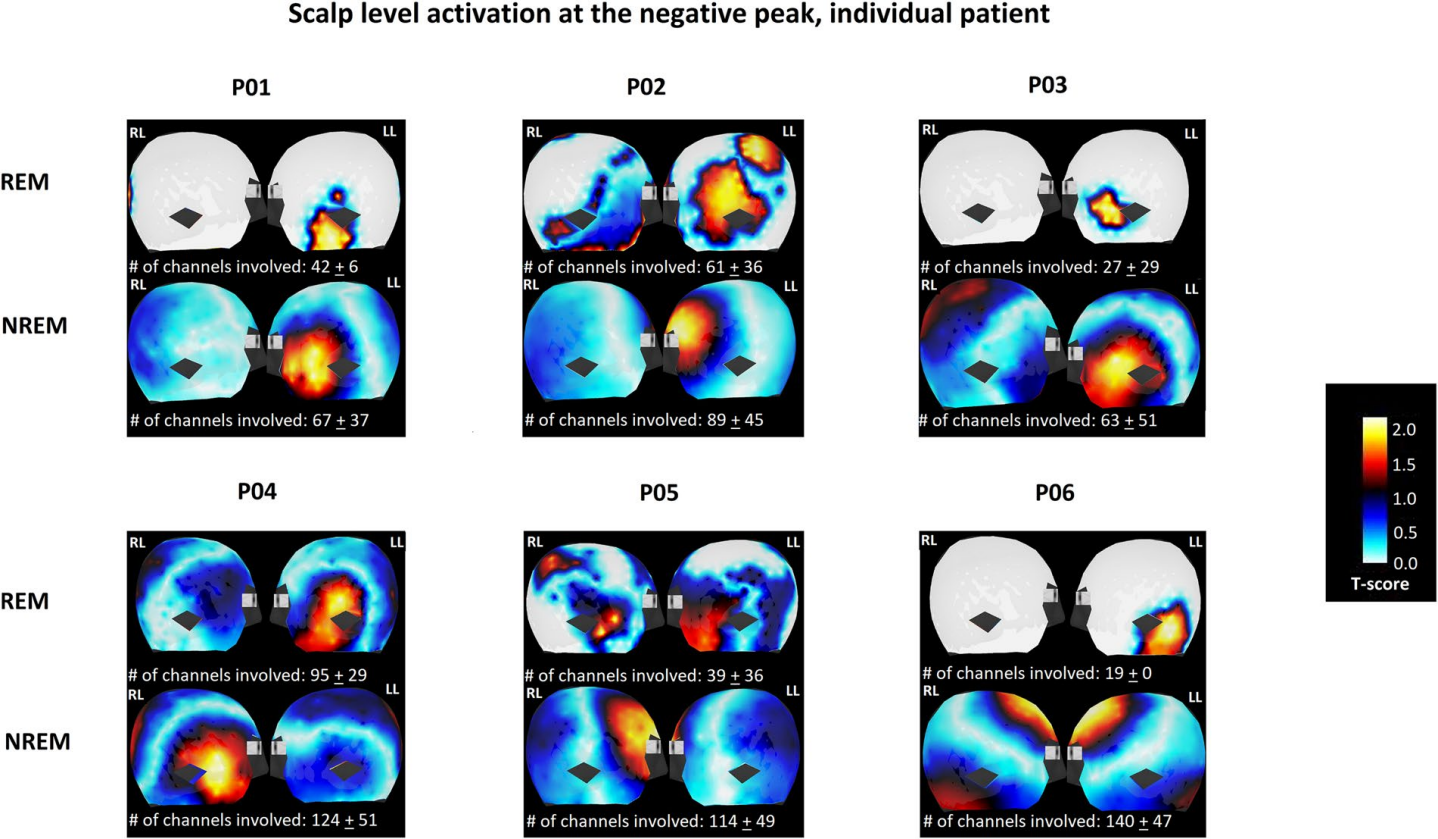
Graphic interpretation of IED scalp level analysis at negative peak. Each subject number is labeled on top of the corresponding figure. For each subject, the left and right lateral view of scalp level activation was demonstrated. The top row represents IED involvement during REM sleep, and the bottom row represents IED involvement during NREM sleep. RL = right lateral; LL = left lateral.

We compared both spike duration and spatial extent (the number of significant time points and of scalp electrodes involved) of IEDs during REM sleep compared to NREM sleep. First, to provide a comprehensive assessment of the overall activity triggered by IEDs, a period between −100 ms to 500 ms around spike negative peak was analyzed. Group-level analysis demonstrated 64% reduction (p = 5 × 10^−11^) of IED duration **(**Fig. 1a**)** during REM sleep compared to NREM sleep, with individual effect size ranging from 36% to 85%. Group-level analysis also demonstrated 32% reduction (p = 2 × 10^−5^) of IED spatial extent (Fig. 1b**)** during REM sleep compared to NREM sleep, with individual effect size ranging from 12% to 74%. Second, we also quantified differences in spatial extent at the spike negative peak. Group analysis at the negative peak demonstrated 53% reduction of IED spatial extent (p = 0.01) during REM sleep compared to NREM sleep **(**Fig. 1c), with individual effect size ranging from 23% to 86%. Numeric values of both group level and individual subject analysis were included in Supplementary Table S2, and individual subject result was plotted in Supplementary Fig. S1. Figure 2 displays topographies of scalp areas activated at spike peak during REM sleep and NREM sleep.

### Source level analysis

**Figure 3:**
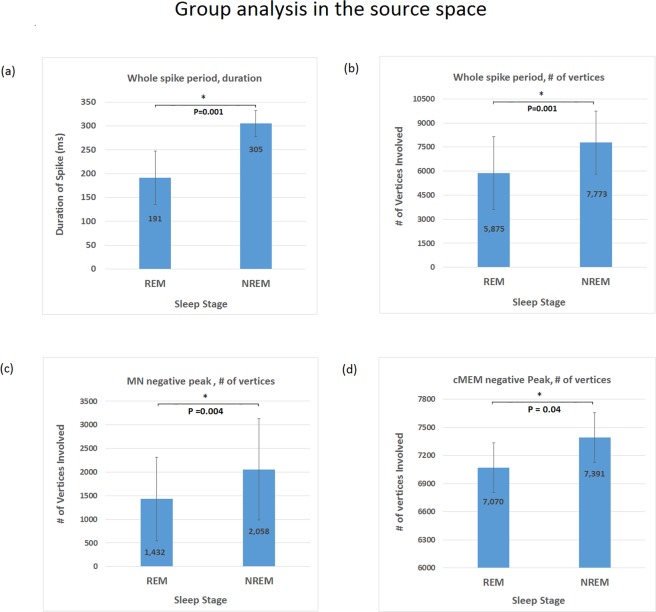
Source localization analysis of IED at different sleep stage: asterisk is applied for result that reached statistical significance. (**a**) IED duration based on source level analysis of the whole spiking period using MN estimate, with shorter duration (p = 0.001) during REM sleep. (**b**) Number of vertices on the cortex involved during IED based on source level analysis of the whole spiking period using MN Estimate, with less channels involved (P = 0.001) during REM sleep. (**c**) Number of vertices on the cortex involved at negative peak based on source level analysis using MN Estimate, with less channels involved (p = 0.005) during REM sleep. (**d**) Number of vertices on the cortex involved at negative peak based on source level analysis using cMEM Estimate, with less channels involved (p = 0.04) during REM sleep.

Because source reconstruction analysis is most often used for pre-surgical work up in clinical practice, we aimed to replicate our results in source space. To do so, we first applied Minimum Norm (MN) imaging^23^, a commonly used method. Similar to scalp analysis, a period between −100 ms to 500 ms was first analyzed to assess overall activity related to IEDs. Group-level analysis demonstrated 37% reduction (p = 0.001) of IED duration **(**Fig. 3a**)** during REM sleep compared to NREM sleep, with individual effect size ranging from 12% to 55%. Group-level analysis also demonstrated 24% reduction (p = 0.001) of IED spatial extent **(**Fig. 3b**)** during REM sleep compared to NREM sleep, with individual effect size ranging from 11% to 55%. Group analysis at the negative peak demonstrated 30% reduction (p = 0.005) of IED spatial extent during REM sleep compared to NREM sleep **(**Fig. 3c), with individual effect size ranging from 8% to 75%. In a second step, we also wished to confirm our results using coherent Maximum Entropy on the Mean (cMEM)^24^ because this method has been shown to account more precisely for the spatial extent of a cortical source. Similar to results obtained with MN, group analysis at the negative peak using cMEM demonstrated 4.3% spatial extent reduction (p = 0.04) of IED spatial extent (Fig. 3d**)** during REM sleep compared to NREM sleep, with individual effect size ranging from 0.3% to 13.7%. Group level and individual subject values are included in Supplementary Table S3. Individual subject results are plotted in Supplementary Fig. S2.

### Comparison with clinical ictal onset zone

**Figure 4:**
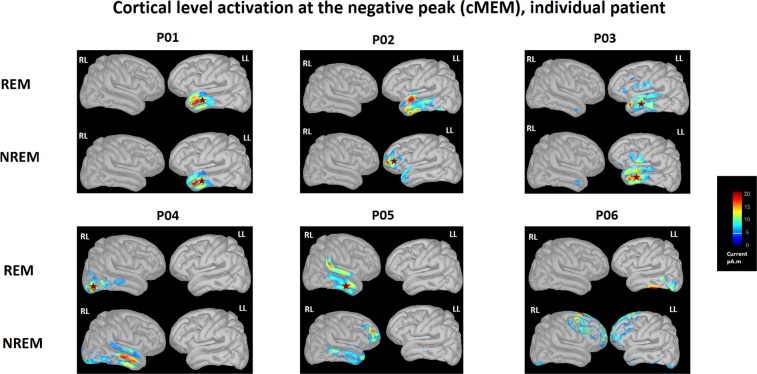
Graphic interpretation of IED source level analysis using cMEM at negative peak. Each subject number is labeled on top of the corresponding figure. For each subject, the left and right lateral view of the cortical reconstruction was demonstrated. The top row represents IED involvement during REM sleep, and the bottom row represents IED involvement during NREM sleep. Asterisk indicates IED with consistent localization as ictal recording obtained using 10–20 system during the same EMU stay. RL = right lateral; LL = left lateral.

To further validate the clinical significance of differences between REM sleep and NREM sleep, we compared the location of source reconstruction results for IEDs obtained in both sleep stages to the patients’ clinical seizure onset zone. A board certified epileptologist (M.B.) reviewed EEG recording during the same EMU stay to extract clinical seizure ictal onset zones, given intracranial recording is unavailable for any of the patients. Lobar correspondence with IEDs source reconstruction results was then qualitatively assessed. Figure 4 shows cortical areas significantly involved at the spike negative peak during REM sleep and NREM sleep for each subject. The spatial distribution of IEDs during REM sleep in subjects 1, 3, 4 and 5 showed consistent localization as the ictal recording during the same EMG stay (4/6 subjects, shown with a *), and for IEDs obtained during NREM sleep, only subjects 1, 2 and 3 (3/6 subjects) showed localization consistent with the ictal recordings.

## Discussion

This report is the first quantitative group study investigating the potential clinical significance of differences in HD-EEG patterns of REM sleep versus NREM sleep IEDs, both at the scalp level and in source space. Our results suggest that IEDs during REM sleep are shorter in duration, involved a more spatially restricted area, and showed a higher concordance with clinical ictal onset zone. These results were consistent both at the group level and at the level of individual subjects.

Our quantitative, group-level results are in line with previous case report studies suggesting that IED during REM may be restricted to the area of the ictal onset zone^18,21,25^. StereoEEG studies suggested that during phasic REM sleep, IED appear to be more suppressed^14^. Even though the amount of IED is reduced during REM sleep compared to NREM sleep, REM sleep IED may tend to be more lateralized to the side of the ictal onset zone^26^.

Our study is the first to directly compare IEDs during REM sleep and NREM sleep after HD-EEG ESI, in source space. Previous topographic characterization of IEDs during NREM sleep showed propagation of IED outside EZ, with a consistent involvement of the medical temporal lobe^27^. Such result is consistent with the observed broader field of NREM sleep IEDs seen in our study. While previous studies have mostly utilized MN for source localization (possibly given its robustness against errors in skull conductivity^28^), more recent work has suggested that cMEM provides source imaging that best reflect the true extent of cortical sources, and is most consistent with EZ^29^. Both MN and cMEM methods showed consistent results in our study, providing evidence that IED during REM sleep were more localized.

The observation reported in our study could be explained by the change of discharge synchronicity during different sleep stages. As demonstrated in previous research, agents that synchronize electric discharges results in increased seizure activities^30^. Conversely, agents that desynchronize electric discharges results in restricted propagation of EEG discharges^31,32^. During NREM sleep, cells discharge synchronously, which augment the spatial propagation of post-synaptic responses^33^. As a result, these changes result in increased cortico-cortical evoked potential (CCEP) and similarly, more widespread IEDs beyond the EZ^22,34^. In contrast, neurons discharge asynchronously during REM sleep^35^, resulting in cortical desynchronization, which may restrict the propagation of IEDs^21,25,36,37^. Similar observation is also supported in high frequency oscillation (HFO) studies, which suggests that a larger field is observed during NREM sleep and a more restricted field is observed during REM sleep^38^.

Unfortunately, none of the 6 patients described here has had invasive monitoring to identify the ictal onset zone. We thus had to define seizure onset zone localization from ictal scalp EEG recordings. The limitation of this approach is that sometimes, scalp EEG cannot show the ictal onset zone due to deep-seated epileptogenic zone. For example, patient 6’s seizures consisted of a whole body feeling of warmth, which could fit with an insular semiology. Although imaging was non-lesional and patient had not undergone invasive monitoring to confirm localization, insular epilepsy remains a strong possibility. This hypothesis could be tested in the future, should the patient opt for phase II EEG monitoring with intracranial EEG. Additionally in patient 2, REM spikes showed left temporal source, but NREM spikes showed left frontal source. This latter finding be in line with a late ictal spread in the scalp EEG ictal towards the left frontal cortex, while the ictal onset zone might be the left temporal, deep seated, which is at times difficult to detect the ictal onset in the scalp EEG, but which REM sleep spike analysis may help to identify. In patient 5, the NREM sleep spike elicited primary activation in the right frontal area with spread to the right temporal lobe. Data area also not consistent with the primary seizure onset zone of the right temporal region as was demonstrated by the REM spike localization. Altogether, these findings suggest that the interpretation of REM sleep versus NREM sleep spike source discrepancies may help to provide useful complementary information when compared to the results coming from ictal clinical EEG recordings. Another limitation of present study is the utilization of templated MRI instead of individualized head models. While the use of a template head model decreases the precision of peak localization from 1 to 2 cm, it was applied similarly in every state and may not account for the observed within-subject differences in extent and duration of IEDs in REM sleep compared to NREM sleep. In addition, the present study is limited by the small number of patients that presented epileptic spikes during both REM sleep and NREM sleep (6/15). Although we employed non-parametric statistics to increase the generalizability of our findings, future studies should study a larger population of patients with focal epilepsy and validate the clinical utility of these findings through a comparison with surgical outcome. Indeed, the irritative zone identified from IEDs captured during REM sleep and NREM sleep should be compared to both the results of invasive monitoring studies as well as to surgical outcomes, because only the latter provides an ultimate validation for the success of localization of the EZ.

## Methods

### Subjects

Fifteen drug-refractory focal epilepsy patients [mean age + standard deviation (SD); 43 + 14, nine females], with focal seizures captured using 10–20 clinical EEG recording that included REM, NREM and awake sleep, were recruited from the epilepsy monitoring unit (EMU) of the University of Wisconsin. The ictal onset zones, which were defined by the ictal recording during the same EMU admission, were localized to either left temporal (n = 4), left frontal (n = 4), right temporal (n = 2), bilateral temporal (n = 2), bilateral frontal (n = 1), bilateral posterior quadrant (n = 1), and right frontal (n = 1) regions on routine scalp EEG. All subjects provided written informed consent before participating in the study. The Institutional Review Board of the University of Wisconsin approved all study procedures. All researches were performed in accordance with relevant guidelines and regulations. Only six subjects who had spikes during both NREM and REM sleep were included in further analysis.

### High density EEG acquisition and preprocessing

256-channel dense array electrodes EEG net (EGI, Electrical Geodesics Inc.) was applied with electrode impedances set below 50 kΩ. It was then applied in between international 10–20 electrode placement with utmost care to avoid bridging. Supplementary Fig. S3 displays a comparison between clinical 10–20 and high-density EEG montages. Twenty-eight to forty-eight hours recordings were performed in patients during the last one to two days of their EMU stays. The cleanest night data was selected for further analysis. Medication was restarted prior to high-density EEG recording. Subjects 1, 2, 3 and 4 were not sleep-deprived. Subject 5 was sleep-deprived the night preceding high-density EEG recording, while subject 6 was sleep-deprived 2 days before recording followed by one recovery night. EEG preprocessing was performed similar to previous work^39–42^. Sleep stages were scored based on the guidelines from AASM scoring manual. Supplemental Table S4 displayed the sleep information for each patient. Epochs of steady stage N2-3 NREM and REM sleep were extracted, filtered through 1–40 Hz filter and down-sampled from 500 Hz to 200 Hz. Semi-automated artifact selection and rejection were performed in order to obtain clean epochs and channel data^40^. Independent component analysis was applied to remove clear sources of physiological noise such as eye movements^42^ in EEGLAB^43^. Board-Certified Epileptologists (R.M. and A.S.) visually detected IEDs during NREM and REM sleep and marked the individual spike locations.

### Scalp-level EEG analysis

EEG was average-referenced using EEGLAB. Epochs containing 1000 ms before and 1000 ms after each spike peak were extracted from the high-density EEG recordings and included in the analysis. Paired 2 tailed t-test was performed between samples from the spiking periods (100 ms before to 500 ms after spike peaks) and baseline (1000 ms before to 300 ms before spike peaks), and alpha level was set at 0.05, corrected for multiple comparison across 256 channels and all time points using Brainstorm^44^. We quantified the duration and number of channels activated during the spiking periods, as well as the number of channels activated at the time of the spike peak, for each spike first. Both spike duration and spatial extent were then averaged across all spikes for each subject during NREM sleep and REM sleep.

To detect group differences between NREM and REM sleep, linear mixed effects model was obtained by using sleep stage as a fixed effect and subject as a random effect. Testing for the significance of models, including spike duration, number of channels involved during each spiking period, and at the negative peak, was done with a likelihood ratio test with comparison to a null model using the Perm Package in R^45^. An additional permutation t-test was performed in each individual patient to assess the reproducibility of intra-subject differences, for spike duration, number of channels involved during spiking period, and at time of spike peak.

### Source space analysis

A template MRI brain provided by Brainstorm software was used for all patients. The forward head model was created by applying 256 leads placement pre-defined in Brainstorm software through the 3-shell sphere option. The source space was constrained to the cortex, which was down-sampled to 15000 vertices.

Source estimation was first achieved through MN imaging. Noise covariance was obtained from the baseline recording (1000 ms before to 300 ms before spike peaks) for each epoch and DC offset was removed. Paired 2 tailed t-test was performed between the current density estimates within the spiking periods and baseline, and alpha level was set at 0.05, corrected for multiple comparison across 256 channels and all time points. The number of vertices involved and duration of the whole spiking period, and numbers of vertices involved at time of negative peak, were obtained for each spike first then averaged across all spikes for each subject during NREM and REM sleep.

Confirmatory analysis using source localization at time of negative peak was also achieved through cMEM. Noise covariance was obtained by applying baseline recording for each epoch and DC offset was removed. Paired 2 tailed t-test was performed between the current density within the spiking periods and baseline, and alpha level was set at 0.01, corrected for multiple comparison across 256 channels and across all time. Number of vertices activated at the time of spike peak was obtained for each spike, and averaged for each subject during NREM and REM sleep.

Similar statistical analysis was performed as described under the scalp level analysis. For source localization utilizing cMEM and MN, linear mixed effects model was obtained, and a likelihood ratio test was constructed to compare duration and number of vertices involved during each spiking period and at the negative peak, during REM vs. NREM. Permutation t-tests were performed in each individual patient to assess the reproducibility of intra-subject differences.

Graphic interpretation of the IED spatiotemporal distributions were obtained using brainstorm software at both scalp and source level. Spatiotemporal distribution of IEDs were compared with the seizure onset zone, which were obtained using 10–20 clinical EEG recording during the same EMU admission, given iEEG was not available in our subjects.

## Supplementary information

Supplemental information.

## Data Availability

The datasets generated during and/or analyzed during the current study are available from the corresponding author upon reasonable request.
