# Supplementary material for: Quantitative spatio-temporal characterization of epileptic spikes using high density EEG: Differences between NREM sleep and REM sleep
Source: Sci Rep. 2020 Feb 3;10:1673. doi: 10.1038/s41598-020-58612-4 (PMC6997449; doi:10.1038/s41598-020-58612-4)
Supplement: Supplementary file 1 — Supplemental information. [file 41598_2020_58612_MOESM1_ESM.pdf]

Quantitative spatio-temporal characterization of epileptic spikes using high density EEG: Differences between NREM sleep and REM sleep

Xuan Kang[1] MD; Melanie Boly[1,2] MD, PhD; Graham Findlay[1,2]; Benjamin Jones[1,2]; Klevest Gjini[1] MD, PhD; Rama Maganti[1] MD; Aaron F Struck[1] MD

Individual subject analysis, scalp level

(a)

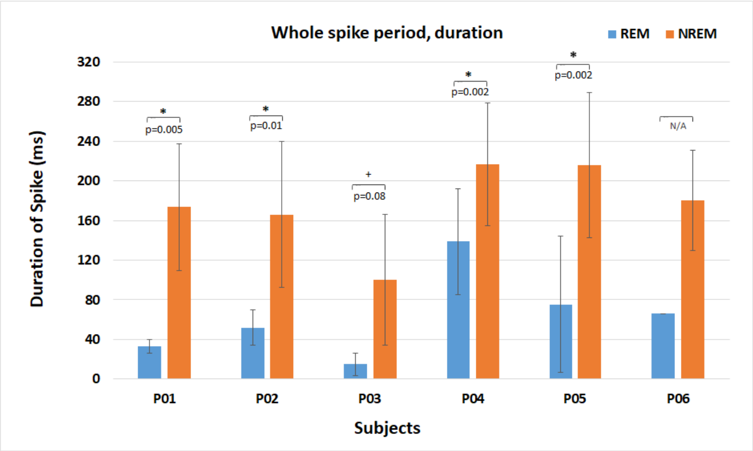

(b)

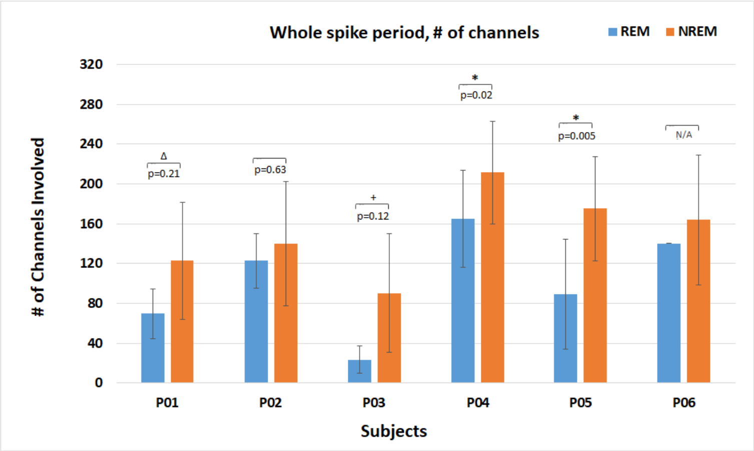

(c)

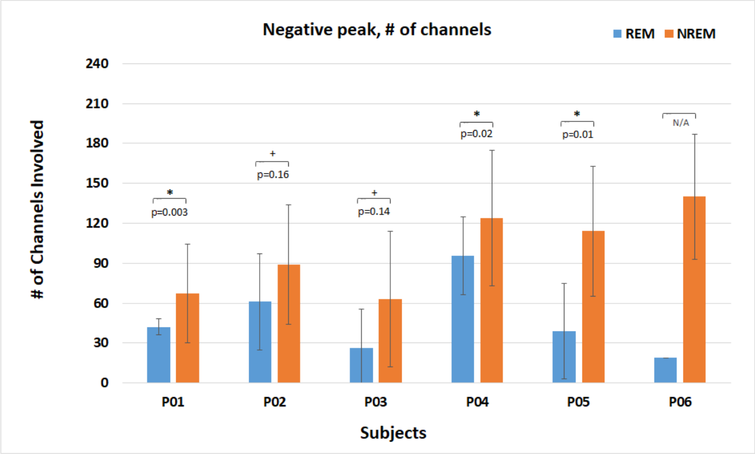

Supplementary Fig. S1. Individual subject analysis at the scalp level. (a): Spike duration based on analysis of the whole spiking period. P01 (p=0.005), P02 (p=0.01), P03 (p=0.08), P4 (p=0.002), P5 (p=0.002). (b) Number of channels involved during IED based on analysis of the whole spiking period. P01 (p=0.21), P02 (p=0.63), P03 (p=0.12), P04 (p=0.02), P05 (p=0.005). (c) Number of channels involved at the negative peak. P01 (p=0.003), P02 (p=0.16), P03 (p=0.14), P4 (p=0.02), P5 (p=0.01)

* indicate result with p value $\leq$ 0.05, + indicate result with p value $\leq$ 0.1, Δ indicate result with p value $\leq$ 0.2

There is no p value for subject 6, given P06 only had 1 spike during REM.

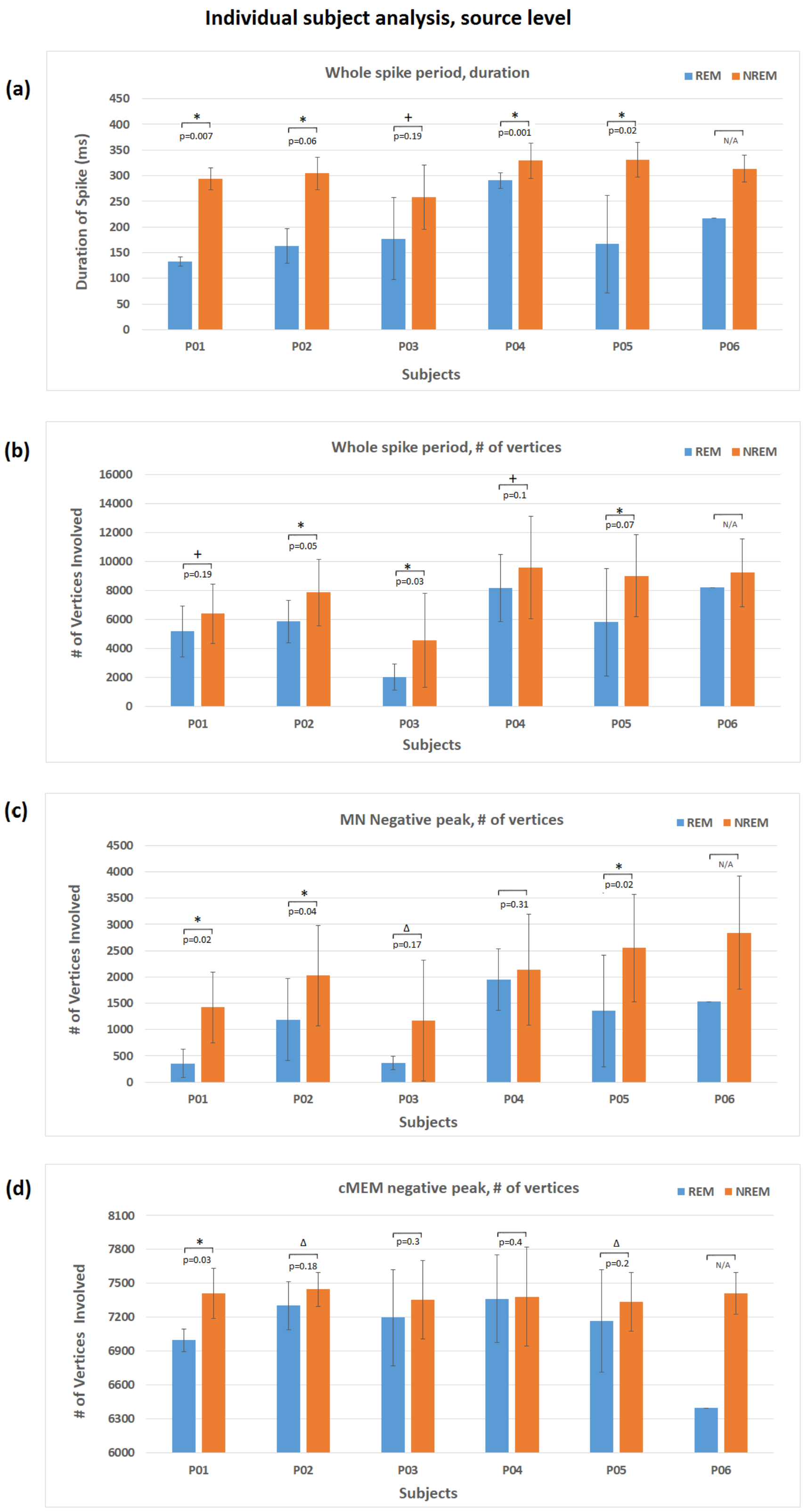

Supplementary Fig. S2. Individual subject analysis at the source level. (a): Spike duration based on analysis of the whole spiking period using MN estimate. P01 (p=0.007), P02 (p=0.06), P03 (p=0.19), P4 (p=0.001), P5 (p=0.02) (b) Number of vertices on the cortex involved during IED based on source level analysis of the whole spiking period using MN Estimate. P01 (p=0.19), P02 (p=0.05), P03 (p=0.03), P4 (p=0.1), P5 (p=0.07) (c) Number of vertices on the cortex involved at negative peak based on source level analysis using MN Estimate P01 (p=0.02), P02 (p=0.04), P03 (p=0.17), P4 (p=0.31), P5 (p=0.02). (d) Number of vertices on the cortex involved at negative peak based on source level analysis using cMEM Estimate. P01 (p=0.03), P02 (p=0.18), P03 (p=0.3), P4 (p=0.4), P5 (p=0.2)
* indicate result with p value $\leq$ 0.05, + indicate result with p value $\leq$ 0.1, Δ indicate result with p value $\leq$ 0.2
There is no p value for subject 6, given P06 only had 1 spike during REM.

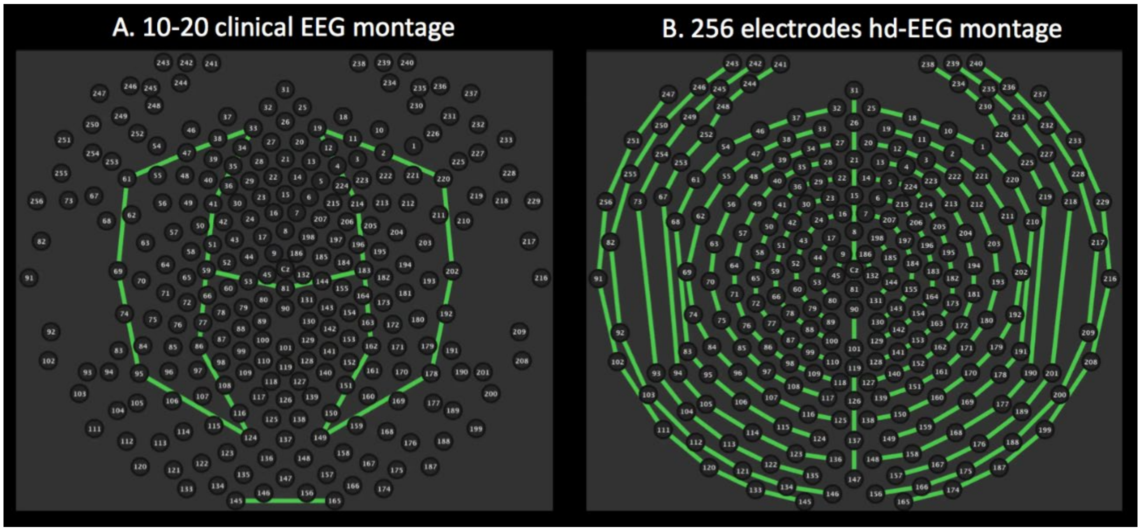

Supplementary Fig. S3. Comparison of spatial coverage between 10-20 and 256 electrodes hd-EEG montages: Clinical 10-20 bipolar EEG montage, overlaid on 256 electrodes location. B. Equivalent 256 electrodes bipolar montage. Numbers correspond to electrodes labels on EGI hd-EEG nets.

	REM	NREM
**Total # of spikes in each sleep stage**	22	186
**# of spikes in individual subject**		
**P01**	2	39
**P02**	3	40
**P03**	2	20
**P04**	10	22
**P05**	4	38
**P06**	1	27

Supplementary Table S1: number of spikes in each data set

	Whole spike period			
		REM	NREM	P-value
**Total**	**Duration (ms)**	63±26	175±65	**5X10^-11**
	**# of channels**	102±28	151±58	**2x10^-5**
**Duration of spike individual subject**	**P01**	33±7	173±64	**0.005**
	**P02**	52±18	166±74	**0.01**
	**P03**	15±11	100±66	**0.08**
	**P04**	139±53	217±62	**0.002**
	**P05**	75±69	216±73	**0.002**
	**P06**	63±0	180±51	N/A
**# of channels individual subject**	**P01**	70±25	123±59	0.21
	**P02**	123±28	140±62	0.63
	**P03**	24±13	90±60	0.12
	**P04**	165±49	211±52	**0.02**
	**P05**	89±55	175±52	**0.005**
	**P06**	140±0	164±65	N/A
	**Negative Peak**			
**Overall**	**# of channels**	47±23	100±47	**0.01**
**# of channels individual subject**	**P01**	42±6	67±37	**0.003**
	**P02**	61±36	89±45	0.16
	**P03**	27±29	63±51	0.14
	**P04**	95±29	124±51	**0.02**
	**P05**	39±36	114±49	**0.01**
	**P06**	19±0	140±47	N/A

Supplemental Table S2: Scalp level analysis: p value reached statistical significance was bolded.
± standard deviation (s.d.)

	**Whole spike period**			
		**REM**	**NREM**	**P-value**
**Total**	**Duration (ms)**	191±56	305±27	**0.001**
	**# of vertices**	5875±2276	7773±1959	**0.001**
**Duration of spike individual subject**	**P01**	133±9	294±22	**0.007**
	**P02**	163±34	304±32	**0.06**
	**P03**	178±80	258±62	0.19
	**P04**	291±15	329±34	**0.001**
	**P05**	167±95	331±33	**0.02**
	**P06**	217±0	314±26	N/A
**# of vertices individual subject**	**P01**	5,185±1,756	6,394±2,061	0.19
	**P02**	5,858±1,451	7,861±2,281	**0.05**
	**P03**	2,034±911	4,558±3,256	**0.03**
	**P04**	8,164±2,296	9,590±3,542	**0.1**
	**P05**	5,809±3,693	9,018±2,839	**0.07**
	**P06**	8,203±0	9,218±2,327	N/A
	**MN negative peak**			
**Total**	**# of vertices**	1,431±884	2,058±1,076	**0.004**
**# of vertices individual subject**	**P01**	357±270	1,423±668	**0.02**
	**P02**	1,188±782	2,026±949	**0.04**
	**P03**	361±128	1,175±1147	0.17
	**P04**	1,954±581	2,134±1,054	0.31
	**P05**	1,358±1,060	2,549±1,024	**0.02**
	**P06**	1,530±0	2,844±1,081	N/A
	**cMEM negative peak**			
**Total**	**# of vertices**	7,070+263	7,391+267	**0.04**
**# of vertices individual subject**	**P01**	6,998+100	7,410+222	**0.03**
	**P02**	7,302+215	7,447+152	0.18
	**P03**	7,197+424	7,355+345	0.3
	**P04**	7,362+388	7,383+436	0.4
	**P05**	7,165+454	7,338+260	0.2
	**P06**	6,396+0	7,410+184	N/A

Supplemental Table S3: Source level analysis: p value reached statistical significance was bolded.
± standard deviation (s.d.)

	TST	REML	SE (%)	%N1	%N2	%N3	%REM
P01	461	414	81	26	66	2	6
P02	243	205	50	21	66	11	2
P03	355	133	57	39	27	26	9
P04	294	290	77	29	58	11	2
P05	470	61	80	39	35	7	20
P06	321	357	59	37	35	16	12

Supplemental Table S4: Sleep information for the patients

TST: total sleep time (in minutes); REML: REM sleep latency; SE: sleep efficiency; %N1: percentage spent in stage N1; %N2: percentage spent in stage N2; %N3: percentage spent in stage N3; %REM: percentage spent in REM sleep
